# Multiple Aspects of PIP2 Involvement in *C. elegans* Gametogenesis

**DOI:** 10.3390/ijms19092679

**Published:** 2018-09-10

**Authors:** Livia Ulicna, Jana Rohozkova, Pavel Hozak

**Affiliations:** 1 Department of Biology of the Cell Nucleus, Institute of Molecular Genetics of the Czech Academy of Sciences, v.v.i., Prague 142 20, Czech Republic; ulicna@img.cas.cz; 2 Department of Epigenetics of the Cell Nucleus, Institute of Molecular Genetics of the Czech Academy of Sciences, v.v.i., Division BIOCEV, Vestec 252 50, Czech Republic; rohozkova@img.cas.cz; 3 Microscopy Centre, Institute of Molecular Genetics of the Czech Academy of Sciences, v.v.i., Prague 142 20, Czech Republic

**Keywords:** nucleus, phosphatidylinositol 4,5-bisphosphate, PPK-1, *C. elegans*

## Abstract

One of the most studied phosphoinositides is phosphatidylinositol 4,5-bisphosphate (PIP2), which localizes to the plasma membrane, nuclear speckles, small foci in the nucleoplasm, and to the nucleolus in mammalian cells. Here, we show that PIP2 also localizes to the nucleus in prophase I, during the gametogenesis of *C. elegans* hermaphrodite. The depletion of PIP2 by type I PIP kinase (PPK-1) kinase RNA interference results in an altered chromosome structure and leads to various defects during meiotic progression. We observed a decreased brood size and aneuploidy in progeny, defects in synapsis, and crossover formation. The altered chromosome structure is reflected in the increased transcription activity of a tightly regulated process in prophase I. To elucidate the involvement of PIP2 in the processes during the *C. elegans* development, we identified the PIP2-binding partners, leucine-rich repeat (LRR-1) protein and proteasome subunit beta 4 (PBS-4), pointing to its involvement in the ubiquitin–proteasome pathway.

## 1. Introduction

Meiotic division is a specific process of gametogenesis, where the DNA from the maternal cell replicates and consequently divides in two turns of cell division, into new haploid germ cells. In *Caenorhabditis elegans*, this includes the pairing of homologous chromosomes, which occurs during the leptotene and zygotene stages of prophase I (transition zone of the germ line). During this highly dynamic process, the two homologous copies of each chromosome find each other within the nucleus through an active search process that enables the chromosomes to distinguish the “self versus non-self” and to assume a side-by-side alignment [1]. The process of pairing is coupled with a synaptonemal complex assembly between the formation of homologs and crossovers (COs), which provides sufficient tension to align the chromosomes on the spindle. As the chromosome pairing, which is a prerequisite for CO formation, is completed by the exit from the transition zone of the gonad, the failure of the COs formation results in randomly segregated chromosomes during the first meiotic division, and subsequent, aneuploidy [2,3].

The processes connected with meiotic progression are complex and involve several regulatory steps that are influenced by the chromatin state. Modifications of DNA are modulating the accessibility of the nucleosomal DNA, which are critical for transcription, replication, recombination, and DNA damage repair [4]. DNA transcription in germ cells is tightly regulated and represents ~21% of the transcription of all known *C. elegans* genes [5], mostly expressing only the proteins involved in or regulating the meiotic progression.

Additionally, changes in the nuclear structure and in both the DNA and RNA polymerases activities can also be caused by nuclear phospholipids [6,7,8,9,10,11]. Previous studies showed that in in vitro models, the addition of positively charged lipids lead to chromosome condensation, while negatively charged lipids caused decondensation [12]. Phosphatidylinositol 4,5-bisphosphate (PIP2) is one of the most studied phosphoinositides. PIP2 localization to plasma membrane, nuclear speckles, small foci in the nucleoplasm, and to the nucleolus were described in mammalian cells [13,14,15,16,17]. It is known that nuclear PIP2 acts as a transcription activator or repressor interacting with various protein complexes of RNA polymerases I and II, or with the histone demethylase [15,16,17,18]. PIP2 regulates the RNA polymerase II transcription apparently by the direct interaction with histone H1 and H3, shielding the positive charges of the histones and thus competing with their ability to bind DNA [19]. Additionally, PIP2 can play a role in RNA polymerase II transcription repression, through binding to the myristoylated transcriptional co-repressor brain acid-soluble protein 1 (BASP1). Toska et al. showed that BASP1 requires PIP2 binding for the recruitment of histone deacetylase 1 (HDAC1) to chromatin, which led to histone deacetylation and thus decreased the promoter accessibility for transcriptional machinery [20].

Phosphoinositides metabolism is very complex thus, to overcome this, a simple organism, *Caenorhabditis elegans*, has been used to investigate the roles of phosphoinositides and its kinases or phosphatases [21,22,23,24,25,26,27,28,29,30]. In *C. elegans*, some enzymes have a unique role, such as Type I PIP kinase (PPK-1 kinase, homolog of PI5PK), thus, we can manipulate specific phosphoinositide species. PPK-1 is a specific kinase that is so far known to be responsible for PIP2 synthesis only. PPK-1 kinase localizes to the plasma membrane and is strongly expressed in the neuronal system of *C. elegans*. The purified PPK-1 kinase is able to generate PIP2 in vitro and the PPK-1 overexpression increases the PIP2 level in vivo [31].

Another described role of PPK-1 is in the *C. elegans* ovulation. PPK-1 localization was observed in somatic tissues, such as neuronal cells [31,32] and also in gonad sheath and distal tip cells, spermatheca, and uterine and vulva muscles [32]. The reduced PPK-1 expression results in the depletion of PIP2, which causes worms sterility, defective ovulation, reduced contractility of sheath cells, and disorganization of myosin filament.

Here, in this study, we observed PIP2 localization in the nuclei in the distal gonad of *C. elegans* hermaphrodite, and in the nuclei of the early embryos. Thus, we focused on the PIP2 functions connected with *C. elegans* chromatin organization and their physiological importance. Interestingly, we discovered that the depletion of PIP2 results in an altered chromosome structure, an impaired chromosome pairing with a consequent defect in crossover formation. Strikingly, these defects were accompanied with increased levels of DNA transcription. As the DNA transcription in the germ cells is tightly regulated [5], the increased localization of RNA polymerase I and II in the germ cell nuclei and the increased 5-Fluorouridine (5-FU) incorporation are probably signals of DNA-damage-driven apoptosis in the *C. elegans* gonad. Moreover, we identified PIP2 interacting proteins in the gonadal nuclei, pointing to its involvement in a ubiquitin–proteasome pathway.

## 2. Results

### 2.1. PIP2 Is Present in C. elegans Prophase I Nuclei and Embryos

**Figure 1:**
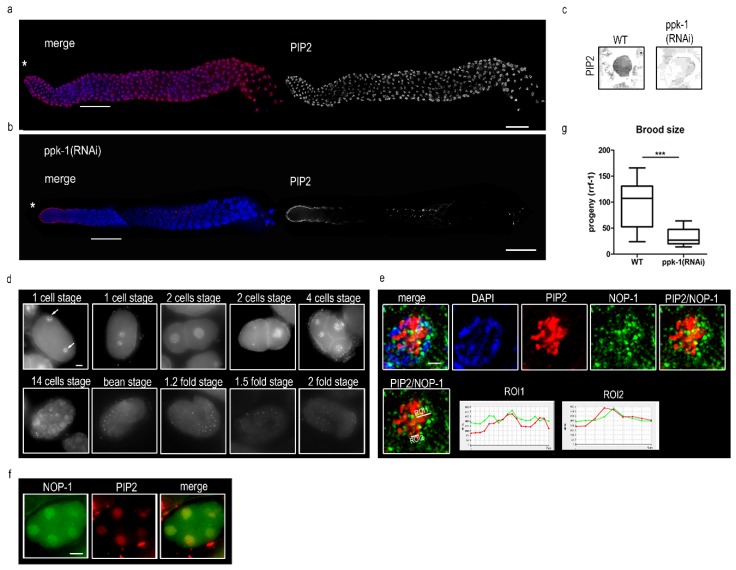
Phosphatidylinositol 4,5-bisphosphate (PIP2) in *C. elegans*. (**a**) Indirect immunofluorescence of PIP2 in a dissected N2 *C. elegans* gonad. Nuclei are visualized by 4′,6-diamidino-2-phenylindole (DAPI); (**b**) indirect immunofluorescence of PIP2 in dissected gonad in ppk-1(RNAi) background. Asterisks mark the distal tip; the transition zone is underlined; the scale bar is 20 µm. The sheath cells’ signal is the unspecific staining of a secondary antibody; (**c**) dot blot detection of PIP2 upon single worm rrf-1(pk1417) whole body lysis in ppk-1(RNAi) or L4440 (WT) background; dot blot I magnified 2×. (**d**) indirect immunofluorescence of PIP2 in embryogenesis of *C. elegans* N2 strain. Arrows point to pronuclei; the scale bar is 5 µm; (**e**) co-localization of anti-PIP2 (red) and anti-NOP-1 (green) in N2 strain germ cell nucleus. Scale bar is 1 µm. Region of interest (ROI1) and ROI2 lines represent the regions of interest for the measured colocalization in pachytene nucleus; (**f**) co-localization of anti-PIP2 (red) and anti-NOP-1 (green) in N2 strain 5-cell stage embryo. Scale bar is 10 µm; (**g**) quantification of brood size, number of fertilized embryos produced by individual hermaphrodites, rrf-1(pk1417) worm in *ppk-1*(*RNAi*) or L4440 (WT) background; (*N* (number of biological replicates) = 3, *n* (number of animals per replica) = 30). Statistical comparisons between the worms were performed using Student *t*-test: n.s.—non-significant; * *p* ≤ 0.05; ** *p* ≤ 0.01; *** *p* ≤ 0.001.

To describe the PIP2 role in *C. elegans*, we decided to investigate its localization in the germinal part of the gonad (prophase I) and in the embryos of the wild type N2 (Bristol) strain. Indirect immunofluorescence using an anti-PIP2 antibody showed the localization of PIP2 in the *C. elegans* germ cells. PIP2 was apparent inside of the prophase I nuclei (transition zone and pachynema), possessing the same intensity and pattern (Figure 1a) along the gonad. It is known that PPK-1 is a PIP2 synthesizing enzyme, thus, the depletion of PIP2 is possible via the knockdown of PPK-1 [31]. After 48 h of RNA interference (RNAi), we observed a dramatic decrease of the PIP2 signal (down to 10%, Figure 1b). The decrease of the PIP2 level after ppk-1(RNAi) was also confirmed by a dot blot detection of PIP2 in a single worm (whole body) lysate (Figure 1c). Next, we observed, by indirect immunofluorescence, that PIP2 is present in the cytoplasm, but also in the nucleus from the 1-cell stage embryo through embryogenesis to the 2-fold stage larvae (Figure 1d), but the intensity of the PIP2 signal decreases after the bean stage of the *C. elegans* embryo. In addition, we observed PIP2 co-localization with NOP-1 (fibrillarin) in the *C. elegans* gonad and embryo (Figure 1e,f), corresponding with the PIP2 nucleolar localization and interaction with fibrillarin in mammalian cells [16,17,33]. Importantly, the observed PIP2 nuclear localisation differs from the PPK-1 localization (Figure S1a,b). The observed nuclear pattern of PIP2 in the meiotic nuclei and embryonic cells recapitulates the known pattern, observed in interphase cells [16,33,34].

### 2.2. Depletion of PIP2 Decreases the Brood Size and Increases Male Incidence

To assess the PIP2 importance in the regulation of the *C. elegans* gametogenesis, we scored the brood size, male incidence, and viability of progeny after the PIP2 RNAi depletion. To differ between the somatic and germ line effect of PIP2, we used the rrf-1(pk1417) worm strain that is sensitive to RNA interference in the germ line, but resistant in the somatic cells [35]. This revealed an original role of PIP2 in the germ cells, additional to the already described function in the soma, in the contractile part of the uterus [31,32]. For the depletion of PIP2 in the rrf-1 strain, we applied an RNAi feeding strategy to decrease the levels of PPK-1 kinase, ppk-1(RNAi). As a control strain, we used a rrf-1(pk1417) *C. elegans* strain fed only with an empty L4440 RNAi vector (wild-type). After 48 h of RNAi via feeding, we observed a decreased brood size (around 60%; Figure 1g) and a higher male incidence (10% per 1350 worms) in the progeny upon the PIP2 depletion ppk-1(RNAi), in comparison to the mock fed control (0.05% per 1200 worms). Also, all of the laid eggs were hatched. These data together suggest that PIP2 is involved in the germ cells processes independently from its functions in the soma.

### 2.3. PIP2 Depletion Causes Aneuploidy and Altered Chromosome Structure in Oocytes

**Figure 2:**
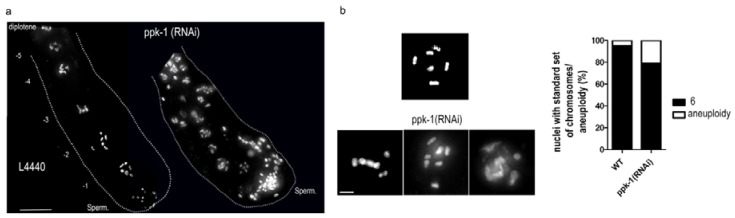
Increased number of DAPI stained bodies. (**a**) DAPI staining of oocytes DNA in the proximal part of dissected rrf-1(pk1417) gonads in ppk-1(RNAi) or WT (L4440) background. Numbers represents the oocytes’ positions. Scale bar represents 15 µm; (**b**) the breadth of oocytes chromosomes phenotypes observed in ppk-1(RNAi) or WT (L4440). The graph represents the % of nuclei with standard chromosome numbers and aneuploidy in ppk-1(RNAi) or WT oocytes (*N* = 3, *n* = 10). Scale bar represents 2.5 µm.

In the next set of experiments, we focused on an explanation for the previously observed increase of the males (X0) and a decrease of the progeny after the PIP2 depletion from the gonad by *ppk-1*(*RNAi*). To address this question, we focused on the nuclei in the proximal part of the *C. elegans* gonad and oocytes [36,37]. For the experiments with ppk-1(RNAi), we again took advantage of the rrf-1(pk1417) *C. elegans* strain, which is RNAi somatic cells insensitive but germ cells sensitive [35]. After 48 h of RNAi treatment, we visualized the DNA by 4′,6-diamidino-2-phenylindole (DAPI) in the dissected gonads. We scored proximal oocyte nuclei that have entered diakinesis. The most proximal oocyte is designated as −1 before ovulation into the spermatheca, where fertilization occurs. We observed that the oocytes in the proximal part of the gonad are dislocalized compared to wild type WT L4440; moreover, the chromosome structure was altered (Figure 2a). We counted the DAPI stained bodies and revealed a presence of univalents (Figure 2b). According to the statistical evaluation, approximately 20% of the ppk-1(RNAi) nuclei have aneuploidy compared with approximately only 3% of the aneuploid nuclei of *C. elegans* WT (Figure 2b).

These are serious phenotypes observed after he PIP2 depletion by ppk-1(RNAi), specifically from the gonad. During the meiotic division, the chromatin state is inevitable to allow for further proper chromosome pairing and segregation. Thus, our observation of the univalent DAPI stained bodies and the altered chromosomes structure in the oocytes led us to speculate that these might be the cause of the previously observed increase of male incidence (X0) in the population and the progeny reduction.

### 2.4. DNA Transcription Increases Upon PIP2 Depletion in Germ Cells Nuclei

**Figure 3:**
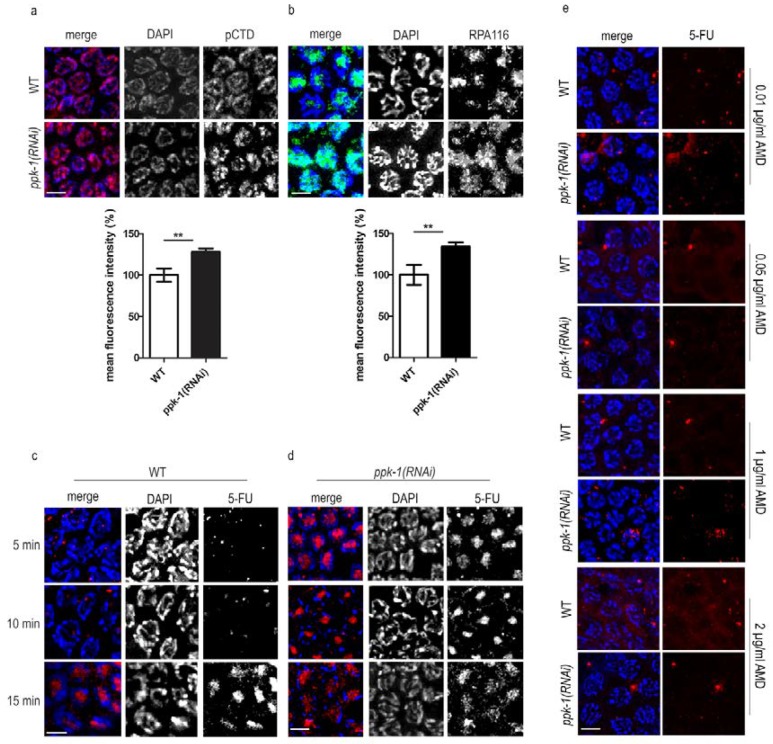
Evidence for increased transcription upon ppk-1(RNAi) in the germ cell nuclei. (**a**) Indirect immunofluorescence of pCTD of RNA polymerase II in ppk-1(RNAi) or L4440 (WT) germ cell nuclei. The graph represents the mean fluorescence intensity measured from *N* = 3 and *n* = 60; (**b**) indirect immunofluorescence of the subunit 116 of RNA polymerase I in ppk-1(RNAi) or L4440 (WT) germ cell nuclei. The graph represents the mean fluorescence intensity measured from *N* = 3 and *n* = 60; (**c**,**d**) indirect immunofluorescence of nascent RNA labelled by 5-Fluorouridine (5-FU) in ppk-1(RNAi) or L4440 (WT) background in the time course (*N* = 3, *n* = 40); (**e**) indirect immunofluorescence of nascent RNA in *ppk-1*(*RNAi*) or L4440 (WT) background upon actinomycin D treatment (*N* = 3, *n* = 30). Scale bars represent 5 µm. Statistical comparisons between the worms were performed using the Student *t*-test: n.s.—non-significant; * *p* ≤ 0.05; ** *p* ≤ 0.01; *** *p* ≤ 0.001.

The germ cell nuclei in prophase I are highly condensed and the transcriptional activity is tightly regulated. After observing the altered chromosome structure, we hypothesized that this might lead to irregularities in chromatin condensation, and we therefore decided to investigate the changes in the transcriptional activity. In this experiment, we again used the rrf-1(pk1417) *C. elegans* strain for the specific depletion of PIP2 from the gonad. We performed indirect immunofluorescence by anti-RNA polymerase II carboxy terminal domain (CTD) subunit phosphorylated at serine 2 antibody (RNA pol II pCTD) and detected an approximately 20% increase in the RNA pol II pCTD signal upon ppk-1(RNAi), compared to the WT gonads (Figure 3a). Next, we performed the same set up of experiments, but investigated the RNA polymerase I localization by the anti-RNA polymerase I (RPA116) antibody (Figure 3b). Also, we observed that RPA116 increased the fluorescent signal by around 30% in the ppk-1(RNAi) gonads compared to WT. These results confirmed the increased presence of RNA polymerase I and the active RNA polymerase II in the germ cells nuclei after the PIP2 depletion from the *C. elegans* gonad.

To examine the change in the transcriptional activity and the level of mRNA, we treated the rrf-1(pk1417) worms with 8 mM 5-Fluorouridine (5-FU), which incorporates into the newly transcribed RNA. We then performed indirect immunofluorescence to visualize the nascent RNA incorporated with 5-FU, using an anti-BrdU antibody that also recognizes 5-FU. We observed that in the ppk-1(RNAi) worms, the labelled nascent RNA was detected already after 5 min of incubation with 5-FU. The labelled RNA was still detectable after 10 and 15 min of incubation with 5-FU in the ppk-1(RNAi) worms. On the other hand, in the WT control gonads, we did not detect the labelled nascent RNA after 5 nor 10 min of incubation with 5-FU. The fluorescent signal of the labelled nascent RNA appeared after 15 min of incubation (Figure 3c,d) in the WT gonads. When we inhibited the transcription by actinomycin D (AMD) in concentrations of 0.01, 0.05, 1, or 2 µg/mL, we detected no 5-FU incorporation after 5 min of 5-FU treatment in the WT nor the ppk-1(RNAi) gonads (Figure 3e). These results strongly suggest that the transcriptional activity measured by the 5-FU incorporation into the nascent RNA was increased in the worms with depleted PIP2.

The obtained data suggest that the increased detection of nascent RNA transcripts is caused by the elevated transcriptional activity in the PIP2 depleted germ cells. Moreover, the elevated transcription is consistent with the increased localization of the RNA pol I and II in the *C. elegans* gonad. All of the data together reveal the PIP2 importance for the transcriptional regulation in the meiotic germ cells’ nuclei of *C. elegans*.

### 2.5. PIP2 Depletion Increases Germ Cells Apoptosis

Because the transcription levels are normally tightly regulated, we hypothesized that the observed abnormally increased localization of RNA pol I and RNA pol II, as well as the increased 5-FU incorporation upon PIP2 depletion (Figure 3), reflect the chromatin changes that may result in a higher apoptosis.

**Figure 4:**
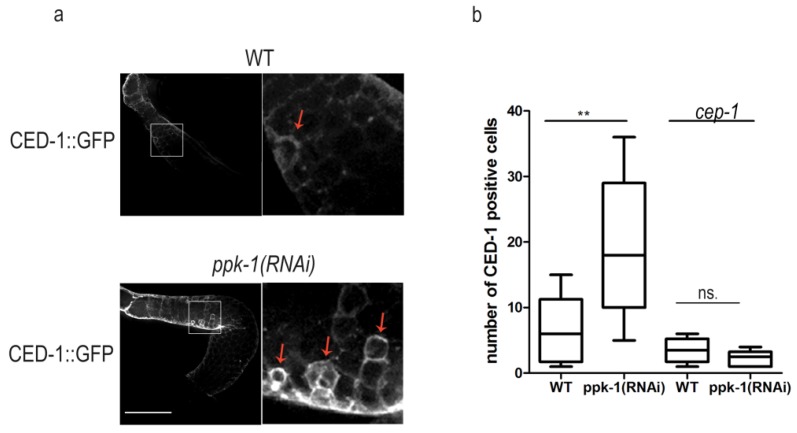
Increased apoptosis upon ppk-1(RNAi) in CED-1::GFP *C. elegans* line. (**a**) CED-1::GFP visualization in ppk-1(RNAi) worms or L4440 (WT) as a control for the background. Red arrows point to the sheath cell localization of CED-1. Scale bar represents 50 µm. (*N* = 3 and *n* = 30); (**b**) graphical representation of the number of CED-1 positive sheath cells in the WT or ppk-1(RNAi) background, with or without CEP-1 (*N* = 3, *n* = 30). Statistical comparisons between worms were performed using the Student *t*-test: n.s.—non-significant; * *p* ≤ 0.05; ** *p* ≤ 0.01; *** *p* ≤ 0.001.

Therefore, we depleted PIP2 from the *C. elegans* CED-1::GFP transgenic line. CED-1 is a phagocytic receptor that localizes to the plasma membrane during apoptosis [38]. We observed an increased CED-1 localization to the plasma membrane in the *ppk-1*(RNAi) worms compared to the control worms. In the ppk-1(RNAi) worms, an average of 19 cells per germ line were positive for CED-1, compared to an average of 7 cells per WT germ line (Figure 4a). Thus, we concluded that the depletion of PIP2 is connected with the increase in apoptosis. To assess whether the observed increase in apoptosis is connected with the DNA damage, we performed ppk-1(RNAi) in the CED-1::GFP *C. elegans* transgenic strain with deleted CEP-1, a p53 mammalian homolog. We scored these worms for the CED-1::GFP distribution. In CED-1::GFP/CEP-1, the mutant worms and an additional PPK-1 knock-down, we detected only background levels of apoptosis. The same background level of apoptosis was noticed in the CED::GFP/CEP-1 mutant worms with PPK-1 present (Figure 4b). These observations suggest that the apoptosis upon PIP2 depletion is caused by DNA damage to CEP-1, in a dependent manner.

Altogether, this supports a hypothesis that the increased localization of RNA pol I and II in the germ cells’ nuclei causes increased apoptosis.

### 2.6. Impaired Chromosome Pairing and Synapsis Defect in ppk-1(RNAi) Worms 

**Figure 5:**
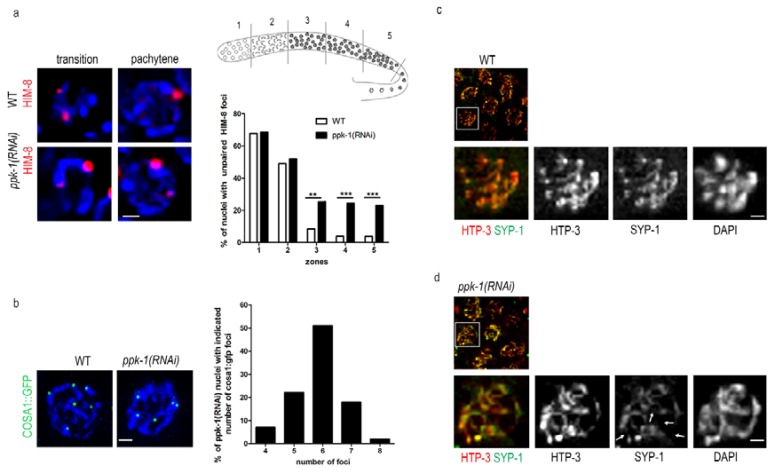
Meiotic defects upon ppk-1(RNAi). (**a**) HIM-8::mCherry visualization in the ppk-1(RNAi) or L4440 background. The graph represents the number of observed HIM-8 foci during prophase I (*N* = 3 and *n* = 30); (**b**) COSA-1::GFP foci vizualization in the ppk-1(RNAi) or L4440 background. The graph represents the % of pachytene nuclei with the given numbers of the COSA-1 foci (*N* = 3 and *n* = 20). Scale bars represent 1 µm; (**c**,**d**) indirect immunofluorescence detection of him 3 paralog (HTP3) and synaptonemal protein 1 (SYP1) in WT or ppk-1(RNAi) background. The arrows point to impaired anti-HTP3 and anti-SYP1 co-localization. Scale bars represent 1 µm. Statistical comparisons between the worms were performed using the Student *t*-test: n.s.—non-significant; * *p* ≤ 0.05; ** *p* ≤ 0.01; *** *p* ≤ 0.001.

It was previously published that the presence of univalents and an altered chromosome structure in the nuclei of the *C. elegans* oocytes is connected with defects in the synaptonemal complex formation and DNA damage [39,40,41]. Here, we already described the altered chromosome structure (Figure 2) that may cause impaired chromosomal pairing and weaken the synaptonemal complex (SC) formation upon PIP2 depletion. Therefore, we decided to investigate the SC formation. We depleted PIP2 by ppk-1(RNAi) in the HIM-8::mCherry *C. elegans* transgenic line, and then visualized the HIM-8 protein, which localizes to the pairing centres at the X chromosome. We observed that, after the PIP2 depletion, the pairing of the X chromosomes was impaired in the pachytene stage of the prophase I, but not in the transition stage. The statistical evaluation of the HIM-8 foci revealed a significant increase (approximately 20%) in the non-paired HIM-8 foci in pachytene in the ppk-1(RNAi) worms, compared to the WT (Figure 5a). The increase of the HIM-8 foci number upon ppk-1(RNAi), but not in the control, supports the PIP2 importance for the proper chromosome pairing. Further on, we investigated the nuclear periphery state by the localization of lamin in the control and ppk-1(RNAi) worms, by indirect immunofluorescence. We did not observe any change in the lamin localization upon the PIP2 depletion from the *C. elegans* gonad (Figure S2a). The unchanged lamin localization confirmed that there are no major changes in the nuclear membrane and the periphery structure.

In the next step, we used indirect immunofluorescence to visualize the synaptonemal complex (SC) formation in the rrf-1(pk1417) *C. elegans* strain. We used anti- him 3 paralog HTP-3 and anti- synaptonemal protein 1 (SYP-1) antibodies to visualize the central and lateral elements of the SC. In the ppk-1(RNAi) worms, we observed an uneven distribution of HTP-3 and SYP-1 with only partial co-localization, which confirmed that the synapsis is not fully formed (Figure 5b). On the other hand, the synapsis in the pachytene nuclei of the WT worms was uniformly extended along the chromosomal axes, with complete co-localization of both SC elements (Figure 5c). These data suggested that the homolog pairs of chromosomes are not aligned properly, thus the SC cannot extend along the full length of the chromosomes, showing the synapsis defect. From these observations, we can conclude that PIP2 is important for chromosome pairing and for the formation of the SC. Furthermore, by investigating the HTP-3 localization in the diakinesis nuclei of WT and ppk-1(RNAi) in the rrf-1(pk1417) strain, we confirmed the presence of chromosome disorganization. HTP-3 has a typical cruciform pattern in the WT worms [42], however, upon PIP2 depletion, HTP-3 is no longer localized to all of the chromosomal arms (Figure S3). These results are apparently connected with the altered chromosome structure (Figure 2), which is the probable cause of improperly paired homologs (lack of HTP-3 at chromosome arms; Figure 5 and Figure S3), and a subsequently increased number of DAPI stained bodies (Figure 2). 

The pairing of homologs and the formation of SC are tightly connected with crossover formation (CO). A disruption of the SC formation between the paired chromosome results in the abrogation of recombination. In the context of previous data, we checked the crossover formation in the *C. elegans*, where the COSA-1 foci correspond to single CO [42]. After the depletion of PIP2 from the *C. elegans* gonad we observed a significant change in the COSA-1::GFP foci compared to the WT gonad (Figure 5d). The statistical evaluation of the COSA-1::GFP foci revealed that approximately 50% of the pachytene nuclei have less (four–five) or more (seven–eight) foci upon ppk-1(RNAi), compared with the WT worms (99%) with six COSA-1::GFP foci per nucleus. These observations might be connected with the chromosome structure and/or the chromosomes’ pairing impairment.

Overall, from this data, we can deduct that PIP2 plays an important role in the chromosome pairing and synaptonemal complex formation, which also affects the crossover formation.

### 2.7. PIP2 Interacts with Proteins Involved in Uubiquitin–Proteasome Pathway

**Figure 6:**
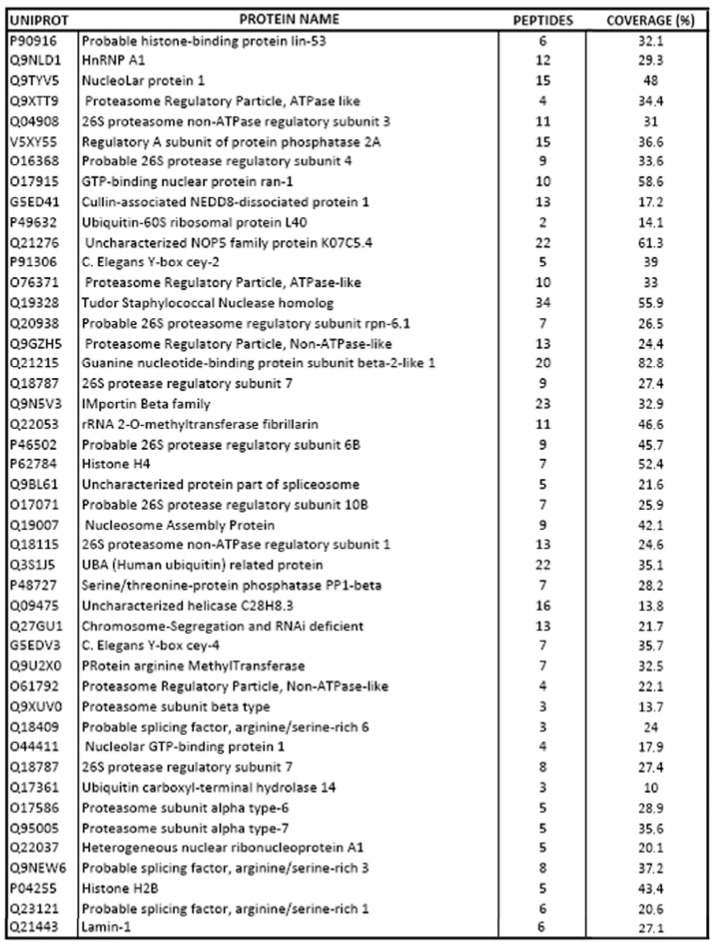
Proteins identified in mas-spectrometry analysis as potential PIP2-binding partners.

To elucidate the molecular mechanism behind the observed effects upon ppk-1(RNAi), we decided to identify the PIP2-binding partners in the *C. elegans* germline. We performed a mass-spectrometry analysis of the material immunoprecipitated either by the anti-PIP2 or by the control mouse anti-IgM antibody from *C. elegans* lysate, prepared from dissected gonads. By comparing the control and anti-PIP2 antibody immunoprecipitates, we identified 45 putative PIP2-binding partners in *C. elegans* (Figure 6), localized either in the cell nucleus and cytoplasm, or exclusively in the cell nucleus.

**Figure 7:**
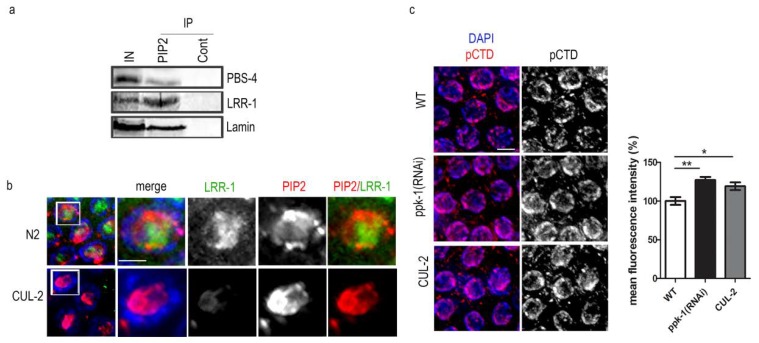
PIP2 interacts with leucine rich repeate protein (LRR-1) and proteasome beta subunit 4 (PBS-). (**a**) Immunoprecipitation by anti-PIP2 and control mouse anti-IgM from *C. elegans* nuclear lysate; (**b**) indirect immunofluorescence of anti-LRR-1 (green) and anti-PIP2 (red) in N2 and CUL-2 background germ cell nuclei. Scale bar represents 2 µm; (**c**) indirect immunofluorescence of pCTD of RNA polymerase II in ppk-1(RNAi) or L4440 (WT) germ cell nuclei. The graph represents the mean fluorescence intensity measured from *N* = 3 and *n* = 30. Scale bar represents 5 µm. Statistical comparisons between the worms were performed using the Student *t*-test: n.s.—non-significant; * *p* ≤ 0.05; ** *p* ≤ 0.01; *** *p* ≤ 0.001.

In the next step, we tested the PIP2 interactions in the nuclear lysate by immunoprecipitation, and we partially confirmed the data from the mass-spectrometry. We endorsed that PIP2 is indeed a binding partner of lamin, proteasome subunit beta 4 (PBS-4), and leucine-rich repeat (LRR-1) protein, which is known as a subunit of the Cullin 2-RING E3 ligase complex (CRL2^LRR-1^) [43,44] (Figure 7a) in *C. elegans*.

Interestingly, LRR-1 co-localizes with PIP2 in the germ cell nuclei (Figure 7b). As the LRR-1 localization depends on cullin-2 (CUL-2) [43], we tested the PIP2 localization in the worms with depleted CUL-2. We did not observe any change in the PIP2 localization, and therefore we conclude that PIP2 interacts with LRR-1 within the same nuclear compartment, but its localization is not dependent on CRL2^LRR-1^ in the germ cell nuclei (Figure 7b).

To focus on the PIP2-LRR-1 complex importance, we investigated the RNA polymerase II localization in the CUL-2 mutant worms and observed significantly increased localization (25% in ppk-1[RNAi] and 20% in CUL-2) compared with the WT worms (Figure 7c).

Here, we identified the PIP2-interacting partners in *C. elegans;* importantly, a number of identified proteins are involved in the ubiquitin–proteasome pathway. Altogether, these results prompt us to suggest that PIP2 might be targeting the RNA polymerase II for degradation by CRL2^LRR-1^.

## 3. Discussion

PIP2 is implicated in various physiological processes therefore it is very challenging to address its individual functions. Most studies on PIP2 and its synthetizing enzymes were performed in mammalian cells, and only a few studies take advantage of the *C. elegans* simpler phosphoinositides metabolism. We decided to address specifically the PIP2 function, and thus we benefit from using *C. elegans* as a model organism for this study. Here, we present that PIP2 influences the chromosomestructure, and is involved in processes such as transcriptional regulation, DNA-damage-driven apoptosis, chromosome condensation with subsequent pairing, and the ubiquitin–proteasome degradation pathway. Based on our data we concluded that the observed *C. elegans* phenotypes might be caused by (i) the altered chromosome structure; (ii) impaired ubiquitin-proteasome pathway; or (iii) disrupted binding of PIP2 to some essential proteins. Based on our data presented here and already published data in mammalian cell lines [16,17,18], we propose that PIP2 is involved in the regulation of the molecular processes at multiple levels, and may be a part of many protein complexes in the *C. elegans* germ cells nuclei.

PIP2 and PPK-1 kinase have important functions in many physiological processes in *C. elegans*, which are crucial in early development [31,32]. Here, we described the PIP2 localization in the *C. elegans* gonad and embryo by an anti-PIP2 antibody directly. PIP2 has a strong nuclear staining during prophase I, which differs from the PPK-1 staining in the whole the gonad. On the other hand, during *C. elegans* embryogenesis, we observed a nuclear, cytoplasmic, and membrane localization of PIP2. Panbianco et al. suggested that PIP2 might be asymmetrically generated in the *C. elegans* embryos, because PPK-1 is enriched at the posterior part of the embryo [45]. However, we did not observe any obvious asymmetrical localization of PIP2 in the embryos, and thus provide more support for another proposal, that PPK-1 might influence the spindle positioning independently of its function as a PIP2 producer. Suggestions about the independent role of PI5PK and PIP2 were previously published by us [18] and Chakrabarti et al. for the regulation of the RNA polymerase I transcription in mammalian cells [46].

We observed an increased incidence of males in the *C. elegans* progeny upon PIP2 depletion (Figure 1g). The *C. elegans* male genotype is represent by only one X chromosome (X0), therefore, we suggest that the increased incidence is dependent on the observed presence of univalent DAPI stained bodies in ppk-1(RNAi) (Figure 2) [47]. Moreover, we observed that the mislocalisation of the oocytes and altered cthe hromosome structure in diakinesis oocytes. The altered chromatin shape was also described after the depletion of ZTF-8 and RAD-51 proteins, and both were shown to be important for the *C. elegans* germ line genomic stability [40,41].

The presence of univalents and the altered chromosome structurein nuclei of *C. elegans* oocytes are connected with defects in the synaptonemal complex formation and DNA damage [40,41]. The depletion of PIP2 resulted in defective SC formation, pointing to the altered chromosome structure in our experiments. This altered chromosome structure probably causes a synapsis defect, which subsequently results in the increased presence of univalents. This is additionally supported by the impaired crossover formation.

The transcription of the germ cell specific, but not somatic genes is ongoing in the germ cell nuclei of *C. elegans*. The germ line transcription represents around ~21% of all known *C. elegans* genes’ transcription, and the ribosomal genes transcription is ~4-fold higher in the germ than in the somatic cell nuclei [5]. Our investigation of the altered chromosome structure after the PIP2 depletion revealed an increased localization of the RNA polymerase I, and a phosphorylated form of RNA polymerase II and a 5-fluorouracil (FU) incorporation in the nascent RNA, both indicat intensified transcription. As transcription is tightly regulated, the increased transcription rate together with the altered chromosome structure is presumably a cause for DNA-damage-driven apoptosis.

In the mammalian cells, it was proven that defects in the nuclear lamina may lead to misshapen nuclei [48]. Another important protein complex is SUN/KASH, which is known to transmit forces from the cytoplasm through the nuclear envelope to the lamina and the chromatin [49]. Penkner et al. showed that the active involvement of the nuclear envelope is inevitable for the homologous chromosome pairing, and that the SUN-1 mutation leads to the disruption of early meiosis chromatin reorganization. On the other hand, they proved that SUN-1 is not essential for SC formation [50]. However, we did not observe any change in the lamin localization or SUN-1 localization (data not shown) upon the PIP2 depletion in the *C. elegans* gonad, thus it is improbable that the observed phenotypes could be caused by cytoplasmic signaling or membranous PIP2.

We hypothesize that these observed impaired processes might originate from the disruption of PIP2 interactions with protein partners. To discover more details, we performed a mass-spectrometry analysis of the immunoprecipitated proteins in complex with PIP2. We identified 45 proteins as potential PIP2 binding partners, where the most striking partners are involved in the ubiquitin proteasome pathway. We showed that PIP2 interacts and co-localizes with the LRR-1 protein in *C. elegans* germ cell nuclei. LRR-1 is a described binding partner of CRL2 E3-ligase [43,44], and is important for proliferation and progression through *C. elegans* germline development. Burger et al. concluded that HTP-3 is likely a direct target for CRL-2^LRR-1^ E3 ligase, as they observed its accumulation in distally located germ cells. We did not observe this accumulation (data not shown). However, they also showed that CRL-2^LRR-1^ acts at multiple levels of germ cell development [44]. Based on different observed phenotypes upon the PIP2 depletion, we hypothesize that the PIP2 interaction with CRL-2^LRR-1^ plays a role at different levels than the CRL-2^LRR-1^ and HTP-3 complex. Probably, the PIP2 interaction targets the CRL-2^LRR-1^ to RNA polymerase II, as we observed an increased RNA pol II localization in the ppk-1(RNAi) and CUL-2 depleted worms [43]. Thus, this supports our suggestion that PIP2 may target CRL-2^LRR-1^ as well as other proteins connected with the disrupted phenotypes upon ppk-1(RNAi). Interestingly, Jungmichel et al. identified Cullin-1 as a possible specific interactor of nuclear PIP2, together with other proteins, such as ubiquitin–protein ligase, ubiquitin-like modifier-activating enzyme, and ubiquitin-associated protein 2 in mammalian cells [51]. PIP2 has multiple protein binding partners (e.g., splicing factors) based on our mass-spectrometry data, and therefore, may play a role at various levels of *C. elegans* development regulation. Consistently, it was shown in mammalian cell lines, that nuclear PIP2 regulates the RNA polymerase I transcription at multiple levels, by the interaction with several proteins [16].

On the other hand, PPK-1 per se may contribute to the regulation of these processes independently of its function as a PIP2 producer, and possibly by the interaction with different protein binding partners, too. Previously, a distinct role of PIP2 and its kinase has been described, based on the binding partners for mammalian cell lines [18,46]. Partially, some of the observed phenotypes might be indirect, caused by the decrease of the second messengers produced by the PIP2 cleavage. However, here, we provide evidence about the PIP2 binding partners that are involved in the regulation of various processes; thus, we are inclined to conclude that the observed phenotypes are connected directly with PIP2. The overall data confirmed the PIP2 involvement and importance in the regulation of crucial processes in the *C. elegans* germ cells.

## 4. Materials and Methods

### 4.1. Strains and Culture Conditions

All of the strains were maintained and cultured under standard conditions at 20 °C using *E. coli* OP50 as a food source [52], except when subjected to RNAi treatment. The used worm strains were obtained from the CGC (Ceanorhabditis Genetics Centre; Minnesota University, Minneapolis, MN, USA): N2 (*C. elegans* var Bristol), NL2098 (rrf-1(pk1417) I.), AV630 (pie-1p::GFP::cosa-1 + unc-119(+)), MD701 (bcIs39 (lim-7p::ced-1::GFP + lin-15(+))), EU640 (cul-2(or209) III.; cultured at 15 °C). We obtained H2B::GFP/HIM-8::mCherry (him-8(tm611) IV; ttTi5605 vv Is17 II; unc-119(ed3) III); H2B::GFP vvIs17(pie-1p::mCherry::him-8::unc-54ter, unc-119(+)) strain from Monique Zetka (McGill University, Montreal, QC, Canada). We obtained CED-1::GFP/CEP-1 (cep-1(ep347) I; bcIs39((lim-7)ced1p::GFP + lin-15(+)) V) strain from Susan Gasser (FMI, Basel, Switzerland).

### 4.2. RNA Interference

For the RNAi of *ppk-1*, we used the full coding sequence of 1836 bp (F55A12.3) from *C. elegans* cDNA. The primers used were as follows: forward 5′-CCCGGGATGGCTTCTCGGTCCAC-3′ and reverse 5′-CCATGGTCAAGCGACAGGTGTGT-3′. The sequence was cloned to a L4440 feeding vector (pPD129.36) [53]. The resulting plasmid was transformed into the HT115(DE3) RNase Ill-deficient *E. coli* strain. Transformed bacteria were spread on an nematode growth medium (NGM) plate with ampicillin (50 mg/mL) and isopropyl β-d-1-thiogalactopyranoside (IPTG; 1 mM). Hermaphrodite worms in the L3/L4 stage were transferred onto the seeded plates and incubated at 20 °C for 48 h. As a mock control, we used empty L4440 plasmid transformed in *E. coli* HT115(DE3) bacteria, and the worms were incubated under the same conditions.

### 4.3. Antibodies 

The following primary antibodies were used in this study: anti-PIP2 mouse monoclonal (Echelon Biosciences Inc., Salt Lake City, UT, USA, clone 2C11, Z-A045), anti-Polymerase I rabbit polyclonal (RPA116; gift from I. Grummt), anti-Polymerase II mouse monoclonal (gift from H. Kimura), anti-BrdU mouse monoclonal (Sigma, St. Louis, MO, USA, 094M4821V), anti-Lamin rabbit polyclonal (Abcam, Cambridge, UK, ab16048), anti-PPK-1 rabbit polyclonal (gift from D. Weinkove), anti-fibrillarin rabbit monoclonal (2639S, Cell signaling, Danver, MS, USA), anti-HTP3 rabbit polyclonal (gift from M. Zetka), anti-SYP-1 mouse monoclonal (gift from M. Zetka), control mouse anti-IgM (Abcam, ab18401), control rabbit anti-IgG (Abcam, ab46540), anti-PBS-4 goat polyclonal (Abcam, ab166792), and anti-LRR-1 rabbit monoclonal (gift from L. Pintard) antibody.

The following secondary antibodies were used in this study: goat anti-mouse IgM (μ-chain specific) antibody conjugated with Alexa Fluor 555 (Invitrogen, Carlsbad, CA, USA, A21426), goat anti-mouse IgG (H+L) antibody conjugated with Alexa Fluor 488 (Invitrogen, A21202), goat anti-rabbit IgG (H+L) antibody conjugated with Alexa Fluor 488 (Invitrogen, A11034), goat anti-rabbit IgG (H+L) antibody conjugated with Alexa Fluor 555 (Invitrogen, A21429), IRDye 680 donkey anti-mouse IgG (H+L) antibody (LI-COR Biosciences, 926-68072), IRDye 800 donkey anti-mouse IgG (H+L) antibody (LI-COR Biosciences Lincoln Nebraska USA, 926-32212), IRDye 800 donkey anti-rabbit IgG (H+L) antibody (LI-COR Biosciences, 925-32213), IRDye 680 donkey anti-rabbit IgG (H+L) antibody (LI-COR, Biosciences, 926-68073), IRDye 800 Goat anti-Mouse IgM (µ chain specific) antibody (LI-COR Biosciences 926-32280), IRDye 800 donkey-anti Goat IgG (H+L) antibody (LI-COR Biosciences, 926-32214), and IRDye 680 donkey-anti Goat IgG (H+L) antibody (LI-COR Biosciences, 926-68074).

### 4.4. Indirect Immunofluorescence, 5-FU Treatment, and Actinomycin D Inhibition

Immunofluorescence staining was performed according to the standard protocol [54]. The images were acquired at the Delta Vision Image Restoration System (Applied Precision, Santa Clara, CA, USA), with stacks of approximately 10 optical sections with 0.3 μm using 60×, 1.2 NA U Plan Apochromat objective (Olympus, Hamburg, Germany). We deconvolved the obtained images using the soft-WoRX 3.0 software (Issaquah Washington, DC, USA). The same imaging conditions were used for the WT and RNAi samples.

The rrf-1(pk1417) strain was subjected to RNAi feeding for 48 h. After 48 h, the adult worms, *ppk-1*(*RNAi*) or WT, were dissected and incubated in 8 mM 5-FU in M9 (22 mM KH_2_PO_4_, 42 mM Na_2_HPO_4_, 86 mM NaCl, 1 M MgSO_4_) for 5, 10, and 15 min. After treatment, the standard protocol for indirect immunofluorescence was followed [54].

For the AMD inhibition, NGM plates for the RNAi knockdown, with addition of different AMD concentrations (0.01, 0.05, 1, and 2 µg/mL) were prepared. After 46 h of RNAi, the rrf-1(pk1417) worms were placed in fresh dishes, to continue the RNAi effect with supplement of AMD. The worms were left on the AMD containing plates for 2 h at 20 °C, then were subjected to 5-FU treatment and, subsequently, indirect immunofluorescence protocol.

### 4.5. Mass-Spectrometry

For the mass-spectrometry (MS) analysis, we used material immunoprecipitated by the anti-PIP2 or control anti-mouse antibody from synchronized adults. The gonads from at least 50 N2 worms were dissected from the body and collected to tubes. The nuclear fraction was prepared according to Singh et al. [55]. After immunoprecipitation, the washed beads were resuspended in 100 mM tetraethylammonium tetrahydroborate (TEAB) containing 1% sodium deoxycholate (SDC). The cysteines were reduced with a 5 mM final concentration of tris(2-carboxyethyl)phosphine TCEP (60 °C for 60 min) and were blocked with a 10 mM final concentration of methyl methanethiosulfonate (MMTS);for 10 min at room temperature). The samples were cleaved on beads with 1 µg of trypsin at 37 °C overnight. After digestion, samples were centrifuged and the supernatants were collected and acidified with trifluoroacetic acid (TFA) to a 1% final concentration. The SDC was removed by extraction to ethyl acetate [56]. The peptides were desalted on a Michrom C18 column. A nano reversed phase column (EASY-Spray column, 50 cm × 75 µm ID, PepMap C18, 2 µm particles, 100 Å pore size) was used for liquid chromatography–mass spectrometry (LC/MS) analysis. The mobile phase buffer A was composed of water and 0.1% formic acid. The mobile phase B was composed of acetonitrile and 0.1% formic acid. The samples were loaded onto the trap column (Acclaim PepMap300, C18, 5 µm, 300 Å wide pore, 300 µm × 5 mm, five cartridges) for 4 min at 15 μL/min. The loading buffer was composed of water, 2% acetonitrile, and 0.1% trifluoroacetic acid. The peptides were eluted with mobile phase B gradient from 4% to 35% B in 60 min. The eluting peptide cations were converted to gas-phase ions by electrospray ionization, and were analysed on a Thermo Orbitrap Fusion (Q-OT-qIT, Thermo). The survey scans of the peptide precursors from 400 to 1600 *m*/*z* were performed at a 120 K resolution (at 200 *m*/*z*), with a 5 × 10^5^ ion count target. The tandem MS was performed by isolation at 1.5 Th with the quadrupole, higher-energy collisional dissociation (HCD) fragmentation with normalized collision energy of 30, and rapid scan MS analysis in the ion trap. The MS 2 ion count target was set to 10^4^ and the max injection time was 35 ms. Only those precursors with a charge state of two–six were sampled for MS. The dynamic exclusion duration was set to 45 s, with a 10-ppm tolerance around the selected precursor and its isotopes. The monoisotopic precursor selection was turned on. The instrument was run at top speed mode with 2 s cycles [57]. All of the data were analysed and quantified with the MaxQuant software (version 1.5.3.8, Martinsried, Germany) [58]. The false discovery rate (FDR) was set to 1% for both the proteins and the peptides, and we specified a minimum length of seven amino acids. The Andromeda search engine was used for the MS/MS spectra search against the *Caenorhabditis elegans* database (downloaded from Uniprot on April 2015, containing 25,527 entries). The enzyme specificity was set as the C-terminal to Arg and Lys, also allowing for the cleavage at the proline bonds and a maximum of two missed cleavages. The dithiomethylation of cysteine was selected as the fixed modification and the N-terminal protein acetylation and methionine oxidation were used as variable modifications. The ‘match between runs’ feature of MaxQuant was used to transfer the identifications to other LC-MS/MS runs, based on their masses and retention time (maximum deviation 0.7 min), and this was also used in the quantification experiments. The quantifications were performed with the label-free algorithms, described recently. The data analysis was performed using Perseus 1.5.2.4 software.

### 4.6. Immunoprecipitation and Dot Blot

The nuclear fraction was prepared according to Singh et al. [55]. For the precipitation, anti-PIP2 (2 µg), control anti-mouse IgM antibody (2 µg), and protein L magnetic beads (50 µL of slurry) were used according to the manufacturer’s protocol (Pierce, Thermo Scientific, Waltham, MA, USA). For the dot blot, the N2 worms were subjected to the *ppk-1*(*RNAi*) or the control RNAi for 48 h. After the RNAi treatment, the single worms were lysed in a lysis buffer (50 mM KCl; 10 mM Tris (pH 8.3); 2.5 mM MgCl_2_; 0.45% NP-40; 0.45% Tween-20) in a total volume of 10 µL. The whole volume was spotted on a polyvinylidene difluoride (PDVF) membrane and the anti-PIP2 antibody was used for detection.

### 4.7. Characterization of Brood Sizes and Embryonic Lethality

The L3–L4 hermaphrodites were individually plated onto NGM plates seeded with *E. coli* HT115(DE3) transformed by L4440 or ppk-1(RNAi) at 20 °C. After 24 h, the adult worms were removed from the plates and the progeny (laid eggs, L1) was counted. Subsequently, we counted the progeny (non-hatched eggs, L1, L2, and L3) after 48 and 72 h. We preformed the experiment on at least for 30 worms in three independent experiments.

### 4.8. Quantitation of COSA-1::GFP, HIM-8::mCherry and RAD-51 Foci

The foci were quantified from deconvolved three-dimensional (3D) data stacks of *C. elegans* WT or *ppk-1*(*RNAi*) germ cell nuclei. We counted the foci in at least 150 germ cell nuclei I, from at least 10 gonads each. The quantification of the RAD-51 foci was performed in the whole gonad composing the mitotic tip to the late pachytene regions [39]. COSA-1::GFP was quantified in late pachytene [42]. HIM-8::Cherry was quantified in transition zone and late pachytene [59]. The statistical comparisons between the genotypes were performed using the Student *t*-test.

### 4.9. Detection of DAPI Stained Bodies in Oocyte Nuclei

The oocyte chromosomes were fixed with 4% formaldehyde and were stained with DAPI [60]. In some of the oocyte nuclei, the individual univalents or bivalents lie too close to each other so as to be resolved unambiguously, thus, this method tends to underestimate the frequency of the achiasmate chromosomes.

## Supplementary Materials

Supplementary materials can be found at http://www.mdpi.com/1422-0067/19/9/2679/s1.
