# Supplementary material for: Multiple Aspects of PIP2 Involvement in C. elegans Gametogenesis
Source: Int J Mol Sci. 2018 Sep 10;19(9):2679. doi: 10.3390/ijms19092679 (PMC6163852; doi:10.3390/ijms19092679)
Supplement: Supplementary file 1 [file ijms-19-02679-s001.pdf]

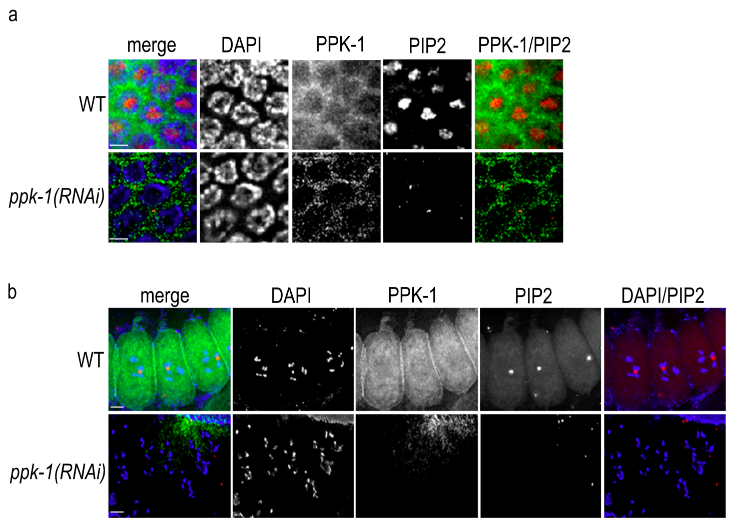

Sup. Figure 1. PIP2 and PPK-1 localization. (a) Indirect immunofluorescence of anti-PIP2 and anti-PPK-1 in WT or *ppk-1(RNAi)* germ cell nuclei background. Scale bar represents 5µm. (b) Indirect immunofluorescence of anti-PIP2 and anti-PPK-1 in WT or *ppk-1(RNAi)* background in oocytes. Scale bar represents 10µm.

a

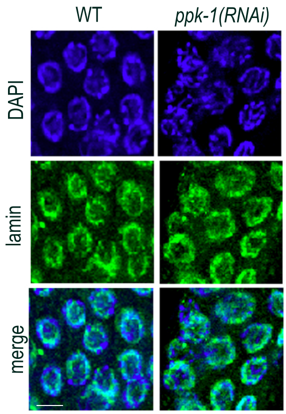

Sup. Figure 2. Lamin localization in L4440 and *ppk-1(RNAi)* gonad. (a) Anti-Lamin indirect immunofluorescence in WT or *ppk-1(RNAi)* background pachytene germ cell nuclei. Scale bars represent 5µm.

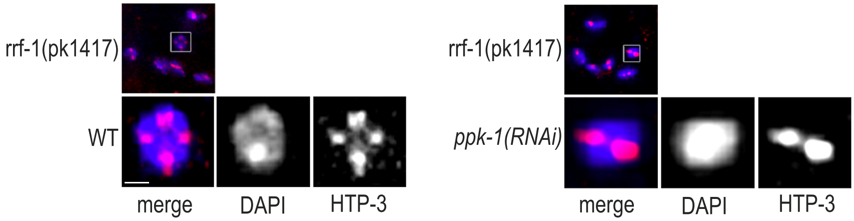

Sup. Figure 3. HTP-3 localization in DAPI stained bodies. Indirect immunofluorescence of anti-HTP-3 in WT *or ppk-1(RNAi)* background. Scale bar represents 2μm.
